# Exploring the Father’s Role in Determining Neonatal Birth Weight: A Narrative Review

**DOI:** 10.3390/medicina60101661

**Published:** 2024-10-10

**Authors:** Alessandro Libretti, Federica Savasta, Anthony Nicosia, Christian Corsini, Alberto De Pedrini, Livio Leo, Antonio Simone Laganà, Libera Troìa, Miriam Dellino, Raffaele Tinelli, Felice Sorrentino, Valentino Remorgida

**Affiliations:** 1 Department of Gynaecology and Obstetrics, University Hospital Maggiore della Carità, 28100 Novara, Italy; federica_savasta@hotmail.it (F.S.); nicosiaanthony@hotmail.it (A.N.); alberto.depedrini@uniupo.it (A.D.P.); libera.troia@uniupo.it (L.T.); valentino.remorgida@uniupo.it (V.R.); 2 School of Gynaecology and Obstetrics, University of Eastern Piedmont, 28100 Novara, Italy; 3 Division of Experimental Oncology/Unit of Urology, URI, IRCCS San Raffaele Hospital, 20132 Milan, Italy; corsini.christian@hsr.it; 4 Department of Urology, Vita-Salute San Raffaele University, 20132 Milan, Italy; 5 Department of Gynecology and Obstetrics, Hopital Beauregard, AUSL Valleè d’Aoste, 11100 Aosta, Italy; lleo@ausl.vda.it; 6 Unit of Obstetrics and Gynecology, “Paolo Giaccone” Hospital, Department of Health Promotion, Mother and Child Care, Internal Medicine and Medical Specialties (PROMISE), University of Palermo, 90127 Palermo, Italy; antoniosimone.lagana@unipa.it; 7 Department of Interdisciplinary Medicine (DIM), Unit of Obstetrics and Gynecology, University of Bari “Aldo Moro”, 70125 Bari, Italy; miriamdellino@hotmail.it; 8 Department of Obstetrics and Gynecology, “Valle d’Itria” Hospital, Martina Franca, 74015 Taranto, Italy; raffaeletinelli@gmail.com; 9 Department of Medical and Surgical Sciences, Institute of Obstetrics and Gynecology, University of Foggia, 71122 Foggia, Italy; felice.sorrentino1983@gmail.com

**Keywords:** anthropometry, birthweight, paternal influence on birthweight

## Abstract

Birth weight, which exhibits variability across different populations, is influenced by a mix of genetic, environmental, and dietary factors originating from both the mother and father. Maternal characteristics, including age, socioeconomic status, prior pregnancies, weight, height, and weight increase throughout pregnancy, have a substantial influence on fetal growth and the health of the infant. On the other hand, the influence of paternal characteristics on the weight of newborns is still not fully comprehended in a consistent manner. Birth weight is an important factor that can help predict various maternal complications, such as the probability of having a C-section, experiencing postpartum hemorrhage or infections. It can also indicate future health challenges like asthma, cognitive impairment, and chronic diseases such as hypertension and diabetes. Nineteen publications were found through a thorough search of the Medline, PubMed, and Scopus databases, which provide insights into how paternal variables contribute to variations in birth weight. Significantly, the age of the father was found to be associated with higher chances of preterm birth and having a smaller size for gestational age in premature infants, while full-term children were more likely to have a larger size for gestational age. In addition, there is a constant correlation between the height of the father and the birth weight of the child. Taller dads are more likely to have babies with a higher birth weight and a lower likelihood of being small for gestational age (SGA). Although there were some discrepancies in the data about the weight and BMI of fathers, it was found that the height of fathers played a significant role in determining the size of the fetus and the weight of the newborn. While there may be differences in the conducted studies, these findings provide valuable insights into the complex connection between parental characteristics and fetal development. This data can be utilized to enhance clinical treatment strategies and enhance our comprehension of outcomes for neonates. Further homogeneous investigations are required to conclusively validate and build upon these findings.

## 1. Introduction

Assessing the weight of newborns is a vital measure of their general health and well-being [1]. Comprehensive previous investigation revealed a strong association between it and both immediate and persistent health problems. Greater birth weights are linked to higher chances of maternal complications, such as the need for C-section delivery, postpartum hemorrhage, uterine rupture, infections, and perineal tears [2,3,4]. Conversely, infants with lower birth weights face the possibility of enduring long-term health issues, including asthma, diminished cognitive abilities, hypertension, type 2 diabetes, and coronary artery disease in their later years [5,6,7].

Environmental factors, such as dietary choices [8], exposure to nicotine [9], consumption of alcohol, and use of drugs, have significant impacts. Furthermore, the interactions among the mother, placenta, and fetus are extremely significant, since alterations in placental development might impede the supply of vital nutrients and oxygen to the fetus [10,11].

The course of fetal growth and development is influenced by an intricate interplay of several elements. Genetic predispositions and parental stature additionally contribute to this process [12,13]. Although the impact of maternal traits on fetal weight [14,15,16] is widely recognized, the influence of paternal variables is still a topic of discussion, despite its acknowledged significance [17,18]. Furthermore, there is a growing body of evidence indicating that some paternal factors may potentially influence the mortality rates and life spans of the descendants [19,20,21,22,23,24,25].

This review analyzes the living literature on the complex association between paternal factors and birth weight, providing a complete insight into this nuanced relationship.

## 2. Methods

The selected technique for this review was determined by the topic’s nature: the authors aimed to emphasize the impact of paternal variables on the offspring’s birthweight. For the sake of completeness, we decided to firstly include all the results without any limitations on publication dates.

A systematic review of peer-reviewed articles was therefore carried out using Medline, PubMed, and Scopus databases but commonly referenced and highly regarded publications from others database, i.e., GoogleScholar, were not excluded. An initial systematic search was conducted using following terms: ‘paternal height’, ‘paternal weight’, ‘paternal BMI’, ‘paternal body mass index’, and ‘paternal anthropometric measurements’ [Title/Abstract].

This first query resulted in the discovery of twenty-five articles. After extracting all the research papers, the writers examined the titles and abstracts, with the goal of removing any duplicate entries. The screening method entailed a meticulous assessment of each study’s abstract to ascertain its pertinence. The authors streamlined the literature pool by eliminating unnecessary repetitions and evaluating relevance. If the significance of an article was not apparent from the abstract, a comprehensive full evaluation of the text was carried out. The review did not include publications written in languages other than English.

Following the initial screening step, the responsibility of assessing the quality of all qualifying studies was assigned to the first four writers, who carried out this task individually to avoid any bias during this first phase. During this stage, the writers thoroughly examined each study, carefully analyzing and classifying every chosen article to comprehensively evaluate the significance, methodology, and results. After, the authors started working closely together to carefully choose the studies that would be included in the review. After individually evaluating each study, they engaged in talks to ensure consensus on the quality and applicability of their findings. During this methodology assessment, a total of 19 publications that precisely addressed the objectives of the study and met the predetermined criteria for inclusion were identified. All these papers were addressing the author’s main question. This was validated by the last author of the present review. The research presented in Table 1 was chosen for its significant contributions to the topic, addressing the main scope of this review. A narrative review was then carried out. This type of review was chosen due to the nature of the topic. The authors carried out the evidence synthesis dividing the main sub-chapters of this review based on the different paternal factors influencing the birthweight, as resulted from the living literature.

## 3. Evidence Synthesis

### 3.1. Paternal Age and Birth Weight

In a comprehensive retrospective study undertaken by Yiting Mao et al. [26], the objective was to investigate the correlation between paternal age and the birth weight of newborns, regardless of whether they were born at term or preterm (less than 37 weeks’ gestation). This comprehensive inquiry utilized data gathered from the International Peace Maternity and Child Health Hospital (Shanghai) over the period of January 2015 to December 2019. An extensive group of 80,811 women was initially examined. After eliminating cases involving twins and those with inadequate paternal age data, the study focused on a substantial sample size of 69,964 cases. To thoroughly examine the influence of paternal age, the study divided the participants into specific age groups using a 10-year range: under 25 years old (n = 752), 25 to 34 years old (n = 48,545), 35 to 44 years old (n = 19,263), and 45 years old or above (n = 1404). The analysis revealed a substantial correlation between paternal age and offspring’s birth weight. Infants born to fathers between the ages of 34 and 44 had a much-increased probability, specifically 23%, of being born with low birth weight compared to infants born to fathers who were younger. In addition, this group exhibited a 7% increased probability of being larger than expected for their gestational age. There was no noticeable difference in the occurrence of newborns being undersized for their gestational age across different paternal age groups. These findings provide insight into the intricate relationship between the age of the father and the weight of the newborn, enhancing our comprehension of this vital element of neonatal well-being.

### 3.2. Paternal Height and Birth Weight

Pritchard and colleagues [27] investigated the paternal height-birth weight hypothesis. The research used data from 7305 newborns, including birth weight, gender, gestational duration, and parental height. This complete dataset came from Aberdeen Maternity Hospital from 1967 to 1971. After removing multiple gestations, unknown gestational ages, extreme gestational lengths, and children delivered to couples with a prior child in the research, 5834 births were selected from the original dataset.

This streamlined sample allowed for a concentrated investigation of paternal height and birth weight. They found a strong link between paternal height and birth weight. Taller dads had heavier babies. Another finding from the research was that spouses in non-manual jobs were taller dads, indicating a relationship between socioeconomic position and paternal height. This suggests, according to the authors, that socioeconomic variables like nutrition and lifestyle may affect the birth weight distribution.

Nasri et al. [28] conducted a retrospective cohort analysis from July 2013 to November 2014 to develop an Iranian birth weight benchmark. Midwives with delivery experience meticulously gathered data from five Iranian hospitals for this challenging project. The information included paternal height and weight, gestational age, parity, and pregnancy complications such diabetes, preeclampsia, and anemia. The research found various factors affecting birth weight through meticulous investigation. Paternal anthropometric parameters indeed showed significant relationships with maternal height and weight. Paternal height correlated with ultrasonography measurements of fetal biometry, including biparietal diameter, cranial circumference, belly circumference, and femur length. These results have consequences for fetal weight and birth weight prediction. Nasri and colleagues stressed the multidimensional nature of birth weight determinants, maternal and other variables, including paternal features. These results highlight the complex interactions that affect infant weight together with the need of considering all variables when setting population-specific birth weight guidelines.

Cawley and colleagues [29] examined the newborns’ weight and parental size. Their study included data from Smethwick, where 1028 youngsters were researched, and Birmingham, where 506 were studied. The data gathering technique recorded both parents’ weight and height, the child’s weight at birth, and three-month post-natal weights up to two years. The investigation revealed newborn’s weight-parental stature relationships. The results showed a strong association between the mother’s height and the child’s birth weight. Interestingly, paternal height had a minor effect on newborn’s weight than mother height. The authors believed these findings showed that the mother’s environment strongly influences prenatal and postnatal baby growth and development. This shows that maternal variables, such as nutritional status, hormonal impacts, and intrauterine variables, shape early baby development trajectories even after birth. This thorough research showed, according to the authors, the complicated relationship between parental factors and newborn’s weight trends, revealing early childhood development processes. The study emphasizes the role of mother health and well-being in treatments to promote optimum newborn’s growth and development.

Prakesh and colleagues [18] reviewed paternal risk factors for low birth weight (LBW), preterm birth (PTB), and small for gestational age (SGA) newborns in 2009. Thirty-six studies examined paternal age, height, weight, birth weight, employment, education, and alcohol intake. Paternal age did not significantly affect PTB or SGA births, according to the review. However, paternal height showed that children born to shorter dads had a birth weight drop of 125 to 150 g. Three of thirty-six studies found an interesting trend: as paternal birth weight increased, infant birth weight increased. Most studies found no significant associations between paternal BMI and birth weight. The research also found a trend: fathers with LBW had higher likelihood of having LBW children. The authors suggested that the occupation of the father and educational achievement may be connected to LBW, warranting additional investigation. However, paternal alcohol usage had contradictory results, highlighting its complexity and influence on delivery outcomes. This thorough analysis endorsed the complex relationship between paternal variables and birth outcomes, suggesting, again, further future research to reduce negative birth outcomes.

Wilcox and colleagues [30] examined 571 partners of unselected women delivering at City Hospital, Nottingham, from August 1992 to February 1993 to determine the effect of paternal size on birth weight, controlling for maternal and fetal factors. When examined individually, paternal height and weight were associated with the offspring birth weight. Multiple regression analysis showed that only paternal height increased birth weight. Paternal height is more likely to represent genetic skeletal potential than obesity risk. To illustrate this link, an average woman’s infant weighed 183 g less if her spouse was shorter (mean −2 standard deviations) than if she was taller (mean +2 standard deviations).

The authors concluded that paternal height may affect birth weight owing to genetics, emphasizing the need of the inclusion of paternal traits in the classification of intrauterine growth retardation or macrosomia. This finding shows the complicated role of genetic variables in fetal development and birth outcomes, affecting clinical practice and perinatal health research.

Takagi and colleagues [31] examined the complex relationship between paternal anthropometric factors including height and BMI and the offspring’s birth weight in a Japanese cohort research from 2011 to 2014. This study was separated by gender to examine gender-specific differences. The study found a strong correlation between increased paternal height and a higher probability of infants being classified as large for gestational age (LGA) and a lower probability of being classified as small/SGA, regardless of gender. Although paternal BMI was relevant, it was less so than height. Additionally, it significantly affected the risk of male newborns being labeled LGA or SGA. The research revealed surprising details about the influence of paternal BMI on newborn’s weight, showing no connection with SGA and only a minor correlation with LGA in female babies. After careful analysis, the authors concluded that paternal height affects the newborn’s birth weight more than BMI. This shows that genetics drive paternal height. The gender-specific effects of paternal BMI on baby weight may be caused by epigenetic events, although the exact processes are unknown. Further research is needed. These results emphasize the relevance of fathers’ characteristics in prenatal care and suggest additional study into paternal effects on the newborn’s health.

### 3.3. Other Paternal Parameters and Birthweight

In a prospective cohort research led by Skaren and colleagues [32], the complicated link between maternal and paternal height and weight and fetal development was examined. The research examined how these parental traits affected fetal femur length (FL) throughout gestational weeks 20 and 30, body length and weight at birth, and postnatal (12 and 24 months) periods in both sexes. The authors recruited 399 families from The Mercy Hospital for Women in Melbourne, Australia, from 2008 to 2009. During pregnancy, mother’s body proportions predicted prenatal FL growth, demonstrating the strong effect of maternal variables on fetal development. On the other hand, fathers’ height alone predicted birth length in female newborns, whereas maternal height did so in male infants. This sex-specific trend remained in a 12-month post-birth weight and length investigation. A transition occurred at 24 months postnatally, when both parents’ body proportions affected offspring’s weight and length in both sexes. The authors hypothesized again that a complex interaction of genetic, epigenetic, and environmental variables caused the link between maternal characteristics and prenatal growth. They stressed the necessity to validate and investigate these results, particularly the sex-based disparities. According to them, the fact that maternal height predicts births length in males and paternal height in girls needs additional study and replication in other groups to determine causes. To summarize, this work heighted the dynamics of fetal and early childhood development and the importance of maternal and paternal traits on offspring’s growth. These results highlight the relevance of parental characteristics in prenatal care and early childhood health treatments, opening the path for further study on intergenerational transmission of qualities affecting child health and development.

Oldereid et al. [33] performed a comprehensive systematic review and meta-analysis of paternal variables on perinatal and pediatric outcomes in 2018. The authors reviewed 13 cohort studies on the effects of paternal BMI, height, and/or weight on obstetric outcomes, notably birth weight (BW). Nine of the thirteen studies examined paternal height and offspring’s BW, consistently finding a link. The link between paternal weight and BMI was complicated. The authors found that paternal BMI correlated with male offspring’s birth weight (BW), biparietal diameter, head size, and pectoral diameter. Three studies found no link between fathers’ BMI and BW, whereas four found no correlation between paternal weight at conception and child BW. According to the authors, paternal height may be associated with offspring’s BW with intermediate confidence.

Morrison et al. [34] investigated paternal variables and the offspring’s birth weight in 1991. The research included birth weight, mother and paternal weight, and height from 5989 couples. The research found that male height significantly affected the birth weight. Birth weight was unaffected by paternal BMI. The research also confirmed the well-established effects of maternal height and weight on fetal weight at delivery, stressing the importance of maternal variables in birth weight outcomes. While paternal height affected birth weight, it had a lesser effect than maternal height and weight. This shows how complicated parental variables affect the offspring’s weight, with maternal traits playing a larger impact. In conclusion, the research shows that the birth weight is affected by several variables, including maternal and paternal effects. These results advance perinatal health and highlight the need for further study into parental impacts on birth weight.

In a prospective study, Wkto et al. [35] examined the relationship between paternal weight, height, and birth weight in 355 middle-class patients with uncomplicated singleton pregnancies at Queen Mary Hospital in Hong Kong from September 1995 to May 1996. Note that maternal data were collected throughout the first trimester and paternal data immediately after childbirth. Birth weight was corrected for gestational age at delivery to maintain data accuracy and reduce pregnancy length differences. In the first univariate analysis, the researchers found statistically significant relationships between paternal and maternal height and weight, maternal pre-pregnancy height and weight, and child-adjusted birth weight. Paternal height, weight, and adjusted birth weight also correlated but not BMI. Multivariate analysis revealed an even more complex result. Only paternal height correlated with adjusted birth weight (*p* < 0.01). The authors concluded that paternal genetic variables may affect fetal development and birth weight. Also, the results of this study illuminate the relationship between parental influences and perinatal outcomes. The authors emphasized the relevance of maternal and paternal variables in understanding fetal development and birth weight dynamics.

### 3.4. Paternal Anthropometry and Birth Weight

Research by Nahum et al. [36] examined the association between paternal traits and birth weight. This large research examined 241 uncomplicated, singleton-term births to see how paternal characteristics including age, height, weight, and BMI affected the birth weight. To fully assess paternal effect on birth weight outcomes, maternal and pregnancy-specific characteristics were included. Both maternal and paternal height predicted term birth weight, with paternal height being somewhat weaker but still significant. For each unit increase in paternal height, the birth weight at term increased by 10 g/cm, demonstrating the strong effect of paternal height on birth weight. The research also showed that dads with heights 2 standard deviations below or above the mean had substantial effects on the offspring’s weight. In contrast to height, paternal age, weight, and BMI did not independently predict birth weight. These data imply that height is a major paternal factor in birth weight, while others may not be.

In a study by Mattsson et al. [37], the authors examined the relationship between parental birth weight and the offspring’s birth weight, considering factors like maternal and paternal age, infant sex, and parity. Using a database of child–mother–father trios from the Swedish Population Register, MBR, and multi-generation register, the researchers examined parental birth weight, gestational age, parity, and maternal smoking habits during pregnancy. The analysis of these interwoven factors revealed compelling insights: a newborn whose father weighed 1500 g less at birth had a birth weight like that of a mother who smoked more than nine cigarettes per day during pregnancy. The study also found a strong link between parental birth weight and baby’s birth weight. In thorough analysis, mother birth weights above 1000 g increased baby’s birth weight by over 245 g, while paternal birth weights over 1000 g increased the newborn’s birth weight by 169 g. Even after controlling for other birth weight parameters, parental birth weight significantly affects offspring’s weight. This emphasizes intergenerational health dynamics by linking parental traits to kids’ development and growth.

Raneen et al. [38] examined how maternal and paternal biometrics affect fetal birth weight in a supplementary prospective study from June 2015 to June 2017. A total of 199 mother–father–neonate trios were examined at Hadassah University Hospital in Jerusalem for anthropometric, gestational, parity, and neonatal sex data. The research found a link between maternal height, pre-pregnancy weight, delivery weight, and neonatal birth weight percentile rank. According to the authors, paternal anthropometry was uncorrelated. The authors proposed an evolutionary interpretation, suggesting that maternal traits may regulate fetal growth in the intrauterine environment to prevent birth-induced complications, while paternal traits may be suppressed to optimize the maternal investment only during pregnancy.

A retrospective study from January to December 2015 at the Department of Obstetrics, Ren Ji Hospital, Shanghai Jiao Tong University, by Xu et al. [39], examined the relationship between preconception paternal body weight and birth weight in 1810 Chinese mother–father–baby trios. A strong correlation was obtained between father’s body weight and birth weight (*p* = 0.02). Even after adjusting for mothers’ age, gestational weight growth, and preconception maternal BMI, this connection remained. Each standard deviation rise in paternal BMI increased neonatal birth weight by 29.6 g. Interestingly, this link was stronger in male children and neonates delivered to overweight moms or those who attained excessive gestational weight. Note that maternal preconception BMI also affected son and daughter birth weight. The results of Chen’s research [41] support the idea that paternal epigenetic information may be passed via the Y chromosome and affect solely male children. This retrospective research concluded how paternal preconception body weight affects neonatal birth weight, revealing epigenetic factors. The findings emphasize the relevance of paternal impacts on maternal and fetal health in agreement with another study [44].

A landmark population-based birth-cohort research by L’Abèe et al. [40] examined parental factors and placenta and infants’ weight in 2011. This study examined the complex interaction between parental traits and newborn’s health variables. A questionnaire was used to analyze parental characteristics, including anthropometric measures, profession, family medical history, lifestyle behaviors including smoking and nutrition, and continuing pharmacological therapy. At birth, clinical data including placental weight and baby’s weight were obtained. The research found a correlation between placental weight and neonate’s weight using multivariate analysis. This highlighted the placenta’s importance in fetal development and growth. This connection was impacted by various maternal and paternal variables. Placental weight was significantly influenced by maternal variables such BMI (*p* < 0.001), birth weight (*p* < 0.001), and diabetes (*p* < 0.001). Paternal variables including BMI (*p* = 0.0034) and birth weight (*p* = 0.001) also affected placental and neonatal weight. The research stressed that maternal variables dominate placental and the newborn’s health, although paternal factors have less of an effect. This stressed the necessity to incorporate maternal and paternal contributions in prenatal care and risk assessment processes to understand newborn’s health outcomes.

### 3.5. Paternal Weight and Birthweight

A retrospective cohort study in China from March to October 2010 by Chen et al. [41] examined the relationship between paternal BMI and fetal growth parameters to determine if paternal characteristics affect neonatal outcomes. The research examined 889 cases, analyzing 492 newborn males and 402 newborn girls. The research examined whether paternal BMI affected the birth weight and fetal ultrasonography data, as well as gender differences. All models showed a substantial association between paternal BMI and birth weight, biparietal diameter, head size, stomach diameter, and pectoral diameter in male progeny. No significant results were obtained for female offspring across these factors. The authors also examined renin activity, aldosterone, cortisol, and fetal glycated serum protein levels in neonatal blood after delivery. This was to determine how paternal BMI affects fetal hormones. They found that newborn male offspring cortisol concentration was significantly associated with paternal BMI, indicating a gender-specific effect on fetal hormone levels. Despite these results, the mechanisms behind these relationships remain unknown. According to the authors, the hypothalamus–hypophysis–adrenal axis may be involved in offspring gender-specific paternal programming, providing an insight into the relationship between paternal influences and newborn’s outcomes.

A cohort of periconceptional couples was recruited by Retnakaran et al. [42] to study the relationship between parental weight before conception and the offspring’s birth weight. This extensive study examined maternal and paternal effects on infant outcomes. At a median of 23.3 weeks before a singleton pregnancy, 1292 couples were examined. Infants were born with a mean birth weight of 3294 ± 450 g, with a significant percentage being either big or small for gestational age. Multivariate analysis of maternal and paternal variables like age, education, smoking status, and pre-conception body mass index (BMI) and gestational factors like duration of gestation, gestational weight gain, gestational diabetes, preeclampsia, and newborn’s sex yielded compelling findings. Each incremental unit in maternal pre-pregnancy BMI increased newborn’s birth weight by 42.2 g, whereas a corresponding rise in paternal BMI added 10.7 g. Maternal pre-pregnancy BMI explained 6.2% of birth weight variation, compared to 0.7% for paternal BMI. Only mothers’ BMI before pregnancy was a significant independent predictor of large-for-gestational-age newborns but paternal BMI did not. In conclusion and according to the authors, fathers’ BMI before conception has a minor effect on newborn’s birth weight, whereas mother pre-pregnancy weight has a major influence. This stresses the role of maternal preconception health in the offspring’s birth weight, requiring more prenatal care examination and intervention.

In 2015, Pomeroy et al. [43] studied 1,041 newborns and their parents’ anthropometric data from the Mater-University of Queensland Study of Pregnancy database in Australia. The researchers examined the links between parental anthropometric factors including height and BMI and newborn’s measures like birth weight, cranial circumference, and trunk and limb length. The study found strong relationships between birth weight and maternal height (*p* < 0.001) and BMI (*p* < 0.001). Additionally, distal limb segments (lower arm and leg) showed strong relationships with paternal height and BMI (*p* < 0.001). Parental anthropometry and newborn’s features interact via genetics, epigenetics, and phenotype. The authors noted that each component’s exact contributions are unknown. They found that maternal anthropometry and head and trunk circumference, together with paternal features, may regulate fetal growth. According to an evolutionary study [43,45,46,47], this adaptation aligns fetal dimensions with the maternal delivery canal to prevent dystocia.

### 3.6. Strength and Limitation

We acknowledge some strengths and limitations of the present study. This review synthesizes data from 19 research studies, providing an extensive viewpoint on the influence of paternal traits on the offspring’s birth weight. Emphasizing paternal height can help underscoring the substantial impact of fathers on fetal growth, particularly on skeletal development, offering important therapeutic insights. We recognize some limitations. There is significant variability in the study included in this paper. This review is hindered by discrepancies in research demographics, methodology, and sample sizes, which restrict the generalizability of the results. Moreover, there could be some confounding factors: variations in ethnicity and other confounding factors may have impacted the findings, hence compromising the consistency of conclusions.

### 3.7. Future Direction for Further Research

Despite the great interest, and although it is well recognized that paternal factors could affect fetal development, growth, and well-being, not many studies have been conducted on this topic. Future research should prioritize performing more uniform studies across many communities to further the understanding of the intricate paternal effects on birth weight. Moreover, additional investigation into genetic, epigenetic, and sex-specific processes is required to explain contradictory results, especially with paternal weight and BMI.

## 4. Conclusions

This evaluation of 19 papers underscores the intricate and multidimensional impact of paternal factors on newborn’s birth weight. A crucial discovery is the substantial influence of paternal height, which was consistently linked to elevated birth weight and prenatal growth, especially via its impact on skeletal development. The interplay between paternal and maternal contributions, especially regarding sex-specific growth patterns, indicates evolutionary adaptations that enhance fetal development. Nevertheless, the information regarding other paternal variables, including weight and BMI, remains incongruous, highlighting the need for further investigation.

## Figures and Tables

**Table 1:** Included studies.

Article	Country	Study Design	Children (n)	Paternal Parameters	Results
Yiting Mao et al., 2019 [26]	China	Retrospective Study	69,964	Paternal Age	Preterm newborns of older fathers have a greater risk of SGA while term newborns of very young or very old fathers have a greater risk of LGA
Pritchard et al., 1983 [27]	Scotland, UK	Cohort study	5834	Paternal height	The standardized birth weights were consistently greater for babies born to women with tall husbands.
Nasri et al., 2015 [28]	Iran	Retrospective cohort study	4994	Paternal height	Strong relationship between paternal height and US parameters (BPD, CC, AC, FL)
Cawley et al., 1954 [29]	UK	Cohort Study	1028	Paternal height	BW of the child increased with increased height of father
Prakesh et al., 2009 [18]	Canada	Systematic Review	ND	Paternal height	Offspring of shorter fathers tended to have, on average, a reduction in birth weight ranging from 125 to 150 g compared to those born to taller fathers.
Wilcox et al., 1993 [30]	UK	Cohort study	571	Paternal height and weight	Increasing paternal height exhibited a significant association with increasing birth weight (*p* = 0.0115)
Takagi et al., 2014 [31]	Japan	Cohort study	33,448	Paternal height	Greater paternal height was associated with increased odds of being large for gestational weight (LGA) and decreased odds of being small for gestational age (SGA) in both female and male neonates
Skaren et al., 2009 [32]	Australia	Prospective cohort study	399	Paternal height	Paternal height uniquely stood out as a predictor of birth length specifically among female infants.
Oldereid et al., 2018 [33]	ND	Systematic review and meta-analysis	ND	Paternal height, weight and BMI	The evidence supporting an association between paternal BMI/paternal weight and offspring BW was deemed to be of lower quality, suggesting little or no discernible correlation.
Morrison et al., 1991 [34]	Australia	Cohort Study	5989	Paternal height and BMI	Paternal height was statistically significant on birth weight (*p* < 0.0007) Paternal BMI did not exhibit a significant effect on birth weight.
WKTo et al., 1998 [35]	China	Cohort study	355	Paternal height, weight and BMI	Paternal height demonstrated a significant correlation with adjusted birth weight (*p* < 0.01), while paternal weight exhibited a marginal correlation (*p* = 0.05)
Nahum et al., 2003 [36]	USA	Cohort study	241	Paternal height	Each unit increase in paternal height was associated with the increase of 10 g/cm in term birth weight. Fathers with heights 2 standard deviations below or above the mean experienced notable changes in offspring birth weight.
Mattsson et al., 2013 [37]	Sweden	Retrospective cohort study	137,538	Paternal birth weight	A birth weight of the father greater than 1000 g corresponded to an increase of 169 g at the birth of the newborn
Raneen et al., 2017 [38]	Israel	Prospective study	199	Paternal weight	No significant correlation with paternal anthropometry.
Xu et al., 2022 [39]	China	Retrospective study	1810	Paternal BMI	Each standard deviation increment in paternal BMI correspond additional 29.6 g increment in neonatal birth weight, especially in male offspring
L’Abèe et al., 2011 [40]	Netherlands	Cohort study	2947	Paternal BMI and birth weight	Paternal factors including BMI (*p* = 0.0034) and birth weight (*p* = 0.001) were found to exert a discernible influence on neonatal weight
Chen et al., 2010 [41]	China	Retrospective cohort study	889	Paternal BMI	In the case of male offspring, all examined models consistently demonstrated a significant correlation between paternal BMI and multiple parameters, including birth weight, biparietal diameter, head circumference, abdominal diameter, abdominal circumference, and pectoral diameter.
Retnakaran et al., 2021 [42]	China	Prospective cohort study	1292	Paternal BMI	Each incremental unit in maternal pre-pregnancy BMI, infant birth weight saw an increase of 42.2 g, while a similar increase in paternal BMI contributed an additional 10.7 g to infant birth weight. Maternal pre-pregnancy BMI accounted for a substantial 6.2% of the variance in birth weight, markedly surpassing the 0.7% explained by paternal BMI.
Pomeroy et al., 2015 [43]	Australia	Prospective cohort study	7223	Paternal BMI	Birth weight displayed marked correlations with maternal height (*p* < 0.001) and BMI (*p* < 0.001). Distal limb segments (lower arm, lower leg) exhibited significant correlations solely with paternal anthropometric measures, including both height and BMI (*p* < 0.001).

## Data Availability

Data gathered for this review are public available to the database cited in the Section 2.

## References

[1] McGuire S.F. (2017). Understanding the Implications of Birth Weight. Nurs. Womens Health.

[2] Oral E., Cağdaş A., Gezer A., Kaleli S., Aydinli K., Öçer F. (2001). Perinatal and Maternal Outcomes of Fetal Macrosomia. Eur. J. Obstet. Gynecol. Reprod. Biol..

[3] Stotland N.E., Caughey A.B., Breed E.M., Escobar G.J. (2004). Risk Factors and Obstetric Complications Associated with Macrosomia. Int. J. Gynecol. Obstet..

[4] Harris R.E. (1970). An Evaluation of the Median Episiotomy. Am. J. Obs. Gynecol..

[5] Mokdad A.H., Ford E.S., Bowman B.A., Dietz W.H., Vinicor F., Bales V.S., Marks J.S. (2003). Prevalence of Obesity, Diabetes, and Obesity-Related Health Risk Factors, 2001. JAMA.

[6] Belbasis L., Savvidou M.D., Kanu C., Evangelou E., Tzoulaki I. (2016). Birth Weight in Relation to Health and Disease in Later Life: An Umbrella Review of Systematic Reviews and Meta-Analyses. BMC Med..

[7] Tirosh A., Shai I., Afek A., Dubnov-Raz G., Ayalon N., Gordon B., Derazne E., Tzur D., Shamis A., Vinker S. (2011). Adolescent BMI Trajectory and Risk of Diabetes versus Coronary Disease. N. Engl. J. Med..

[8] Duttaroy A.K. (2023). Influence of Maternal Diet and Environmental Factors on Fetal Development. Nutrients.

[9] Fernandez-Rodriguez B., Gomez A.R., Jimenez Moreno B.S., de Alba C., Galindo A., Villalain C., Pallás C., Herraiz I. (2022). Smoking Influence on Early and Late Fetal Growth. J. Perinat. Med..

[10] Satterfield M.C., Edwards A.K., Bazer F.W., Dunlap K.A., Steinhauser C.B., Wu G. (2021). Placental Adaptation to Maternal Malnutrition. Reproduction.

[11] Sacks D.A. (2004). Determinants of Fetal Growth. Curr. Diab Rep..

[12] Cassidy F.C., Charalambous M. (2018). Genomic Imprinting, Growth and Maternal–Fetal Interactions. J. Exp. Biol..

[13] Kiserud T., Benachi A., Hecher K., Perez R.G., Carvalho J., Piaggio G., Platt L.D. (2018). The World Health Organization Fetal Growth Charts: Concept, Findings, Interpretation, and Application. Am. J. Obs. Gynecol..

[14] Cogswell M.E., Yip R. (1995). The Influence of Fetal and Maternal Factors on the Distribution of Birthweight. Semin. Perinatol..

[15] Roland M.C.P., Friis C.M., Godang K., Bollerslev J., Haugen G., Henriksen T. (2014). Maternal Factors Associated with Fetal Growth and Birthweight Are Independent Determinants of Placental Weight and Exhibit Differential Effects by Fetal Sex. PLoS ONE.

[16] Swamy G.K., Edwards S., Gelfand A., James S.A., Miranda M.L. (2012). Maternal Age, Birth Order, and Race: Differential Effects on Birthweight. J. Epidemiol. Community Health.

[17] Kim S., Kim M., Oh M.-Y., Seo Y., Yum S.K. (2023). Impact of Increased Paternal Age on Neonatal Outcomes in Very-Low-Birth-Weight Infants. J. Matern.-Fetal Neonatal Med..

[18] Shah P.S. (2010). Paternal Factors and Low Birthweight, Preterm, and Small for Gestational Age Births: A Systematic Review. Am. J. Obs. Gynecol..

[19] Sharma R., Agarwal A., Rohra V.K., Assidi M., Abu-Elmagd M., Turki R.F. (2015). Effects of Increased Paternal Age on Sperm Quality, Reproductive Outcome and Associated Epigenetic Risks to Offspring. Reprod. Biol. Endocrinol..

[20] Barclay K., Myrskylä M. (2018). Parental Age and Offspring Mortality: Negative Effects of Reproductive Ageing May Be Counterbalanced by Secular Increases in Longevity. Popul. Stud..

[21] du Fossé N.A., van der Hoorn M.-L.P., van Lith J.M.M., le Cessie S., Lashley E.E.L.O. (2020). Advanced Paternal Age Is Associated with an Increased Risk of Spontaneous Miscarriage: A Systematic Review and Meta-Analysis. Hum. Reprod. Update.

[22] Urhoj S.K., Andersen P.K., Mortensen L.H., Davey Smith G., Nybo Andersen A.-M. (2017). Advanced Paternal Age and Stillbirth Rate: A Nationwide Register-Based Cohort Study of 944,031 Pregnancies in Denmark. Eur. J. Epidemiol..

[23] Khandwala Y.S., Baker V.L., Shaw G.M., Stevenson D.K., Lu Y., Eisenberg M.L. (2018). Association of Paternal Age with Perinatal Outcomes between 2007 and 2016 in the United States: Population Based Cohort Study. BMJ.

[24] Alio A.P., Salihu H.M., McIntosh C., August E.M., Weldeselasse H., Sanchez E., Mbah A.K. (2012). The Effect of Paternal Age on Fetal Birth Outcomes. Am. J. Mens. Health.

[25] Nybo Andersen A.-M., Urhoj S.K. (2017). Is Advanced Paternal Age a Health Risk for the Offspring?. Fertil. Steril..

[26] Mao Y., Zhang C., Wang Y., Meng Y., Chen L., Dennis C.-L., Sheng J., Wu Y., Huang H. (2021). Association Between Paternal Age and Birth Weight in Preterm and Full-Term Birth: A Retrospective Study. Front. Endocrinol..

[27] Pritchard C.W., Sutherland H.W., Carr-Hill R.A. (1983). Birthweight and Paternal Height. BJOG.

[28] Nasri K., Hantoushzadeh S., Hugh O., Heidarzadeh M., Habibelahi A., Shariat M., Tara F., Kashanian M., Radmehr M., Yekaninejad M.S. (2021). Customized Birthweight Standard for an Iranian Population. J. Matern.-Fetal Neonatal Med..

[29] Cawley R.H., McKeown T., Record R.G. (1954). Parental Stature and Birth Weight. Am. J. Hum. Genet..

[30] Wilcox M.A., Newton C.S., Johnson I.R. (1995). Paternal Influences on Birthweight. Acta Obs. Gynecol. Scand..

[31] Takagi K., Iwama N., Metoki H., Uchikura Y., Matsubara Y., Matsubara K., Nishigori H., Saito M., Fujiwara I., Sakurai K. (2019). Paternal Height Has an Impact on Birth Weight of Their Offspring in a Japanese Population: The Japan Environment and Children’s Study. J. Dev. Orig. Health Dis..

[32] Skåren L., Davies B., Bjørnerem Å. (2020). The Effect of Maternal and Paternal Height and Weight on Antenatal, Perinatal and Postnatal Morphology in Sex-stratified Analyses. Acta Obs. Gynecol. Scand..

[33] Oldereid N.B., Wennerholm U.-B., Pinborg A., Loft A., Laivuori H., Petzold M., Romundstad L.B., Söderström-Anttila V., Bergh C. (2018). The Effect of Paternal Factors on Perinatal and Paediatric Outcomes: A Systematic Review and Meta-Analysis. Hum. Reprod. Update.

[34] Morrison J., Williams G.M., Najman J.M., Andersen M.J. (1991). The Influence of Paternal Height and Weight on Birth-weight. Aust. N. Z. J. Obstet. Gynaecol..

[35] To W., Cheung W., Kwok J. (1998). Paternal Height and Weight as Determinants of Birth Weight in a Chinese Population. Am. J. Perinatol..

[36] Nahum G.G., Stanislaw H. (2003). Relationship of Paternal Factors to Birth Weight. J. Reprod. Med..

[37] Mattsson K., Rylander L. (2013). Influence of Maternal and Paternal Birthweight on Offspring Birthweight—A Population-based Intergenerational Study. Paediatr. Perinat. Epidemiol..

[38] Raneen A.S., Lina D.S., Safrai M., Matan L., Porat S. (2022). Is Birthweight Influenced Equally by Maternal and Paternal Anthropometry?. J. Matern.-Fetal Neonatal Med..

[39] Xu R., Zhao W., Tan T., Li H., Wan Y. (2022). Paternal Body Mass Index before Conception Associated with Offspring’s Birth Weight in Chinese Population: A Prospective Study. J. Obs. Gynaecol..

[40] L’Abée C., Vrieze I., Kluck T., Erwich J.J.H.M., Stolk R.P., Sauer P.J.J. (2011). Parental Factors Affecting the Weights of the Placenta and the Offspring. J. Perinat. Med..

[41] Chen Y.-P., Xiao X.-M., Li J., Reichetzeder C., Wang Z.-N., Hocher B. (2012). Paternal Body Mass Index (BMI) Is Associated with Offspring Intrauterine Growth in a Gender Dependent Manner. PLoS ONE.

[42] Retnakaran R., Wen S.W., Tan H., Zhou S., Ye C., Shen M., Smith G.N., Walker M.C. (2021). Paternal Weight Prior to Conception and Infant Birthweight: A Prospective Cohort Study. Nutr. Diabetes.

[43] Pomeroy E., Wells J.C.K., Cole T.J., O’Callaghan M., Stock J.T. (2015). Relationships of Maternal and Paternal Anthropometry with Neonatal Body Size, Proportions and Adiposity in an Australian Cohort. Am. J. Phys. Anthr..

[44] Pembrey M.E., Bygren L.O., Kaati G., Edvinsson S., Northstone K., Sjöström M., Golding J. (2006). Sex-Specific, Male-Line Transgenerational Responses in Humans. Eur. J. Hum. Genet..

[45] Trevathan W., Rosenberg K. (2000). The Shoulders Follow the Head: Postcranial Constraints on Human Childbirth. J. Hum. Evol..

[46] Benjamin S.J., Daniel A.B., Kamath A., Ramkumar V. (2012). Anthropometric Measurements as Predictors of Cephalopelvic Disproportion. Acta Obs. Gynecol. Scand..

[47] Kjærgaard H., Dykes A.K., Ottesen B., Olsen J. (2010). Risk Indicators for Dystocia in Low-Risk Nulliparous Women: A Study on Lifestyle and Anthropometrical Factors. J. Obs. Gynaecol..

